# Cognitive-Enhancing Effect of Marine Brown Algae-Derived Phenolics through S100B Inhibition and Antioxidant Activity in the Rat Model of Ischemic Stroke

**DOI:** 10.3390/md22100451

**Published:** 2024-10-01

**Authors:** Jurairat Khongrum, Pratoomporn Yingthongchai, Suriya Tateing, Pratchaya Kaewkaen

**Affiliations:** 1 Multidisciplinary Research Institute, Chiang Mai University, Chiang Mai 50200, Thailand; jurairat.kh@cmu.ac.th (J.K.); pratoomporn.y@cmu.ac.th (P.Y.); 2 Functional Food Research Center for Well-Being, Multidisciplinary Research Institute, Chiang Mai University, Chiang Mai 50200, Thailand; 3 Department of Plant and Soil Sciences, Faculty of Agriculture, Chiang Mai University, Chiang Mai 50200, Thailand; suriya.t@cmu.ac.th; 4 Animal Cognitive Neuroscience Laboratory (ACoN), Faculty of Education, Burapha University, Chon Buri 20131, Thailand

**Keywords:** S100B protein, marine algae, *Sargassum polycystum*, cognition

## Abstract

Cognitive impairments are frequently reported after ischemic strokes. Novel and effective treatments are required. This study aimed to develop a functional ingredient obtained from marine algae and to determine the effect of the extract on antioxidative stress, as well as neuroprotective effects, in a rat model of MCAO-induced ischemic stroke. Among the selected marine algal extracts, *Sargassum polycystum* displayed the highest total phenolic content and antioxidative potential, and was subsequently used to evaluate cognitive function in rat models of ischemic stroke. The *S. polycystum* extract, administered at doses of 100, 300, and 500 mg/kg BW, significantly improved cognitive function by enhancing cognitive performance in the Morris water maze and novel object recognition tests. Biochemical changes revealed that providing *S. polycystum* increased the activities of SOD, CAT, and GSH-Px by 52.48%, 50.77%, and 66.20%, respectively, and decreased the concentrations of MDA by 51.58% and S100B by 36.64% compared to the vehicle group. These findings suggest that *S. polycystum* extract may mitigate cognitive impairment in ischemic stroke by reducing oxidative stress and inhibiting S100B expression, thus highlighting its potential as a functional ingredient for drugs and nutraceuticals aimed at neuroprotection.

## 1. Introduction

Currently, cerebrovascular disease is recognized as a stroke resulting from pathological disorders in blood vessels, which is a major cause of severe morbidity and mortality. According to several sources of evidence, acute ischemic stroke occurs as a result of multiple factors, such as disruption of the blood-brain barrier and vascular changes that reduce blood flow to the brain [1,2]. Intensive scientific research has reported vascular changes resulting in reductions in blood flow, which are linked to the development of vascular cognitive impairment [3]. Furthermore, pre-clinical and clinical studies have revealed that the imbalances in oxidative stress have been identified as critical contributors to the pathophysiology of stroke-induced cognitive deficits, where excessive free radicals cause neuronal damage [4,5]. Additionally, the expression S100B protein, a small regulatory calcium-binding protein, serves as a biomarker for detecting early stages of Alzheimer’s disease, and plays a vital role in the development of degenerative disease and brain injury [6,7]. Several studies have reported the potential neuroprotective effects of maintaining S100B protein levels, which may help safeguard against neuronal damage and dysfunction [8], thereby reducing brain tissue death, and enhancing cognitive function [9]. Since vascular cognitive impairment associated with ischemic stroke is a complex disorder with no current satisfactory treatment, therapeutic approaches with neuroprotective and regenerative effects have gained much attention.

Recent studies have emphasized the potential of marine algae as a source of bioactive compounds with antioxidant properties [10] and neuroprotective effects [11], making them as promising candidates for the prevention of cognitive decline and neurodegenerative diseases. Specifically, *Sargassum polycystum*, a brown seaweed, is rich in polyphenols, carotenoids, and polysaccharides. Moreover, *S. polycystum* is notable for its high phenolic content [12], which has demonstrated anti-inflammatory, antioxidant, antidiabetic, and neuroprotective activities in preclinical models of neurodegenerative diseases [13]. Phenolic compounds, such as phenolic acid, which are abundant in *S. polycystum* [14], are recognized for their ability to cross the blood-brain barrier, reduce reactive oxygen species (ROS), and exert neuroprotective effects [15].

Therefore, we hypothesized that a seaweed extract containing phenolic compounds could potentially protect against cognitive impairment caused by cerebral ischemic stroke. Since no prior data is available, this study is aimed to assess the total phenolic content and antioxidant properties of nine seaweeds, composed of red seaweed, brown seaweeds, and green seaweeds. Subsequently, we evaluated the potential effects of the selected seaweeds on enhancing cognitive function in a rat model of ischemic stroke induced by permanent middle cerebral artery occlusion (MCAO). Furthermore, this work investigates the potential underlying mechanisms, with a specific emphasis on alleviating oxidative stress and decreasing S100B levels.

## 2. Results

### 2.1. Characterization of Selected Seaweeds on Phenolic Compounds and Antioxidant Activity

Nine seaweed extracts, including *Caulerpa lentillifera*, *Chaetomorpha antennina*, *Enteromorpha prolifera*, *Sargassum polycystum*, *Padina minor*, *Padina australis*, *Gelidium pusillum*, *Jania rubens*, and *Gelidiella acerosa*, were evaluated for their total phenolic content, radical scavenging activity, and reducing power. The results are shown in Table 1.

The content of phenolic compounds in the extracts revealed that *S. polycystum*, *P. minor*, and *P. australis* had the highest phenolic contents (92.25 ± 3.26, 54.27 ± 1.59, and 76.29 ± 2.42 mg GAE/g, respectively), while the lowest phenolic contents were found in *C. lentillifera*, *G. pusillum*, and *C. antennina* (3.42 ± 0.25, 15.95 ± 1.38, and 16.42 ± 1.21 mg GAE/g, respectively).

The highest DPPH radical scavenging potency was observed in *P. minor* (205.32 ± 2.01 µg/mL), followed by *P. australis* (159.42 ± 1.83 µg/mL) and *S. polycystum* (124.36 ± 5.04 µg/mL). In contrast, *C. lentillifera*, *C. antenina*, and *G. acerosa* exhibited the lowest radical scavenging activity against DPPH (32.71 ± 0.43, 45.26 ± 3.51, and 56.42 ± 5.62 µg/mL, respectively).

The reducing power of selected seaweeds also showed that the greatest reducing antioxidant power compared to standard ascorbic acid was expressed in *S. polycystum*, followed by *E. prolofera*, and *P. australis* (63.24 ± 2.79, 63.00 ± 6.92, and 35.82 ± 2.54 mg AAE/g extract, respectively). Conversely, *C. lentillifera*, *G. acerosa*, and *J. Rubens* showed the smallest reducing power (9.56 ± 4.23, 16.34 ± 3.02, and 12.08 ± 2.54 mg AAE/g extract, respectively).

### 2.2. Gallic Acid Content in S. polycystum Extracts by HPLC Analysis

**Figure 1:**
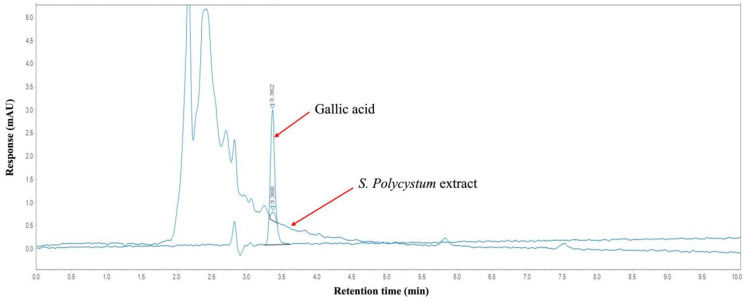
High-performance liquid chromatography (HPLC) chromatogram showing the separation profile of standard gallic acid and *S. polycystum* extract, used to compare retention times and assess the presence of phenolic compounds.

Based on in vitro results, *S. polycystum* was selected to study its effect on cognitive function in rat models of ischemic stroke because it contained the highest phenolic compound content and exhibited strong antioxidant properties, including radical scavenging activity and reducing power. Phenolic compounds are important phytoconstituents due to their scavenging ability, which is attributed to their hydroxyl groups. HPLC analysis confirmed the presence of gallic acid, a powerful natural phenolic compound with potent antioxidant and neuroprotective properties, in *S. polycystum* extracts. Figure 1 shows the HPLC chromatograms obtained from the gallic acid standard and *S. polycystum* extracts. The amount of gallic acid in the *S. polycystum* extract was found to be 114.02 ± 0.34 μg/g extract.

### 2.3. Effect of S. polycystum on Cognitive Functions by Morris Water Maze

**Figure 2:**
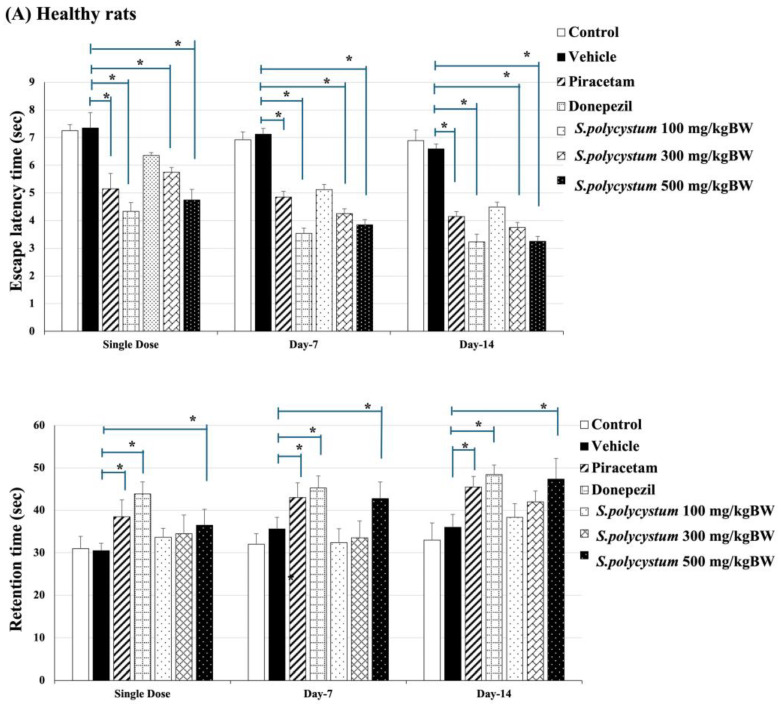

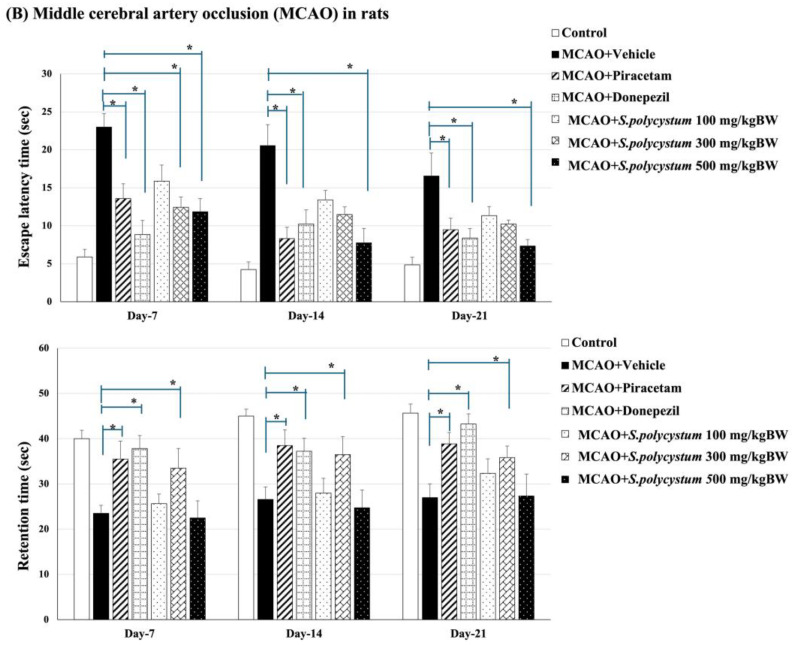
Effect of *S. polycystum* extract on spatial memory in rats, as assessed using the Morris water maze test. Each column and bar represent a mean value ± S.E.M. (n = 8/group). * *p*-value < 0.05 compared to the vehicle group. (**A**) Cognitive-enhancing effect of the extract by MWT test in healthy rats; (**B**) Cognitive-enhancing effect of the extract by MWT test in the MCAO stroke model.

The results are shown in Figure 2. In healthy conditions (before Rt. MCAO surgery), rats that received the vehicle displayed a significantly increased escape latency, but decreased retention time (*p* < 0.001 for both measures, compared to the control group). Rats that received a *S. polycystum* extract at doses of 300 and 500 mg/kg BW showed a significant decrease in escape latency time (*p* < 0.05, all; compared to the vehicle group). Additionally, only the high dose of *S. polycystum* extract significantly increased retention time (*p* < 0.05, compared to the vehicle group) on days 1, 7, and 14. Under cognitive impairment conditions (after Rt. MCAO surgery), rats that received *S. polycystum* extract at doses of 300 and 500 mg/kg BW showed a significant reduction in escape latency time on day 1 (*p* < 0.05, all; compared to the vehicle group) whereas only the high dose showed a significant reduction throughout the study period (*p* < 0.05, all; compared to the vehicle group). On the other hand, the dose of 300 mg/kg BW showed a decreased retention time throughout the study period (*p* < 0.05, all; compared to the vehicle group). Interestingly, both positive treatments, including piracetam and donepezil, displayed a significant reduction in escape latency and a significant improvement in retention time (*p* < 0.05 for both measures, compared to the vehicle group).

### 2.4. Effect of S. polycystum on Cognitive Functions by Novel Object Recognition Test (NOR)

**Figure 3:**
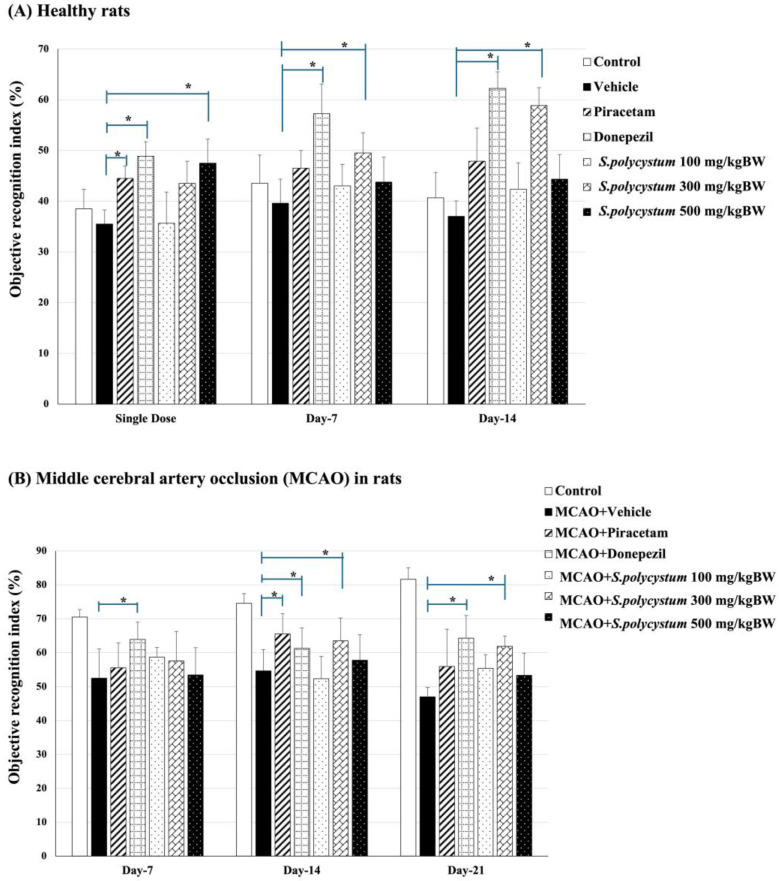
Effect of *S. polycystum* extract on novel object memory in rats, as assessed using the novel object recognition test. Each column and bar represent a mean value ± S.E.M. (n = 8/group). * *p*-value < 0.05 compared to the vehicle group. (**A**) Cognitive-enhancing effect of the extract by NOR test in healthy rats; (**B**) Cognitive-enhancing effect of the extract by NOR test in the MCAO stroke model.

We evaluated the percentage of the object recognition index under two conditions: a healthy condition and an ischemic stroke condition. The data is shown in Figure 3. Under the healthy condition, we performed NOR test on single dose, day 7, and day 14. The rats that received an *S. polycystum* extract at a dose of 500 mg/kg BW saw a significantly enhanced recognition index in the single-dose test (*p* < 0.05, compared to the vehicle group). On day 7 and day 14, only a medium dose (300 mg/kg BW) showed an increase in the objective recognition index (*p* < 0.05 for all; compared to the vehicle group). After Rt. MCAO implementation, we found that rats that received a *S. polycystum* extract at a dose of 300 mg/kg BW saw an increase of the percentage for the objective recognition index on day 14 and day 21, compared to the vehicle group (*p* < 0.05 for all; compared to the vehicle group). In addition, rats that received the positive drugs, including piracetam and donepezil, also showed a significant increase in the objective recognition index throughout the study.

The results showed that administration of *S. polycystum* extract led to reduced escape latency and increased retention time in the Morris water maze test, as well as improved recognition indices in the Novel Object Recognition test, indicating improved cognitive performance in a rat model of ischemic stroke.

### 2.5. Effect of S. polycystum Extract on the Oxidative Stress Status

**Figure 4:**
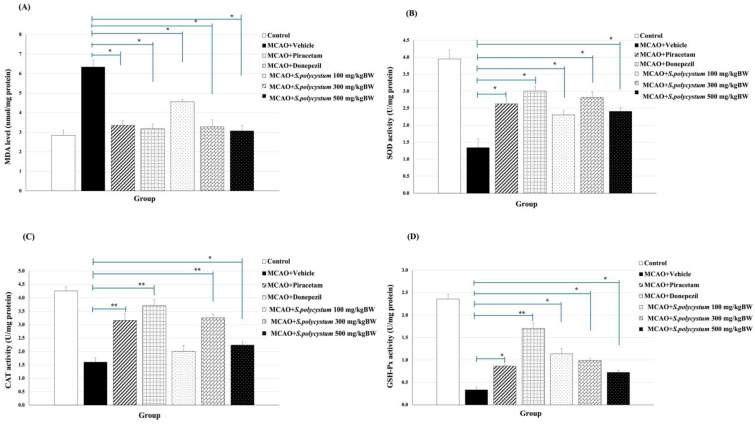
Effect of *S. polycystum* extract on oxidative stress status. Each column and bar represent a mean value ± S.E.M. (n = 8/group). ** *p*-value < 0.01, * *p*-value < 0.05, compared to the vehicle group. (**A**) Effect of the extract on the level of malondialdehyde (MDA) in the hippocampus; (**B**) Effect of the extract on the activity of superoxide dismutase (SOD) in the hippocampus; (**C**) Effect of the extract on the activity of catalase (CAT) in the hippocampus; and (**D**) Effect of the extract on the activity of glutathione peroxidase (GSH-Px) in the hippocampus.

Because the hippocampus plays a crucial role in the regulation of cognitive function, and its pathology is associated with manifestations of cognitive impairment, the effect of *S. polycystum* extract on oxidative stress status in the hippocampus was determined. The data are shown in Figure 4. Rt. MCAO-exposed rats that received the vehicles saw a significant decrease in SOD, CAT, and GSH-Px activities, but saw an increase in MDA level (*p* < 0.01 for GSH-Px and CAT, *p* < 0.05 for SOD and MDA; compared to the control group). The low dose of the extract significantly reduced the MDA level, and increased SOD, and GSH-Px activities (*p* < 0.05, all; compared to the vehicle group). The medium dose of the extract significantly decreased the MDA level and increased SOD, CAT, and GSH-Px activities (*p* < 0.05, 0.05, 0.01, and 0.05, respectively, compared to the vehicle group). The high dose of the extract significantly decreased the MDA level, and increased SOD, CAT and GSH-Px activities (*p* < 0.05, all; compared to the vehicle group). Additionally, treatment with piracetam and donepezil resulted in a significant reduction in MDA levels and an increase in SOD, CAT and GSH-Px activities.

Therefore, this study demonstrated that *S. polycystum* extract increased the activities of antioxidant enzymes (SOD, CAT and GSH-Px) and reduced the levels of MDA, a biomarker associated with oxidative stress damage. The increase in SOD, CAT and GSH-Px activities helps to alleviate oxidative stress in the hippocampus, a critical brain region responsible for cognitive functions. Thus, these biological changes are consistent with the hypothesis that the seaweed extract could help prevent and mitigate cognitive decline in ischemic stroke by enhancing the antioxidant defense mechanisms.

### 2.6. Effect of S. polycystum Extract on Serum S100B Concentration

**Figure 5:**
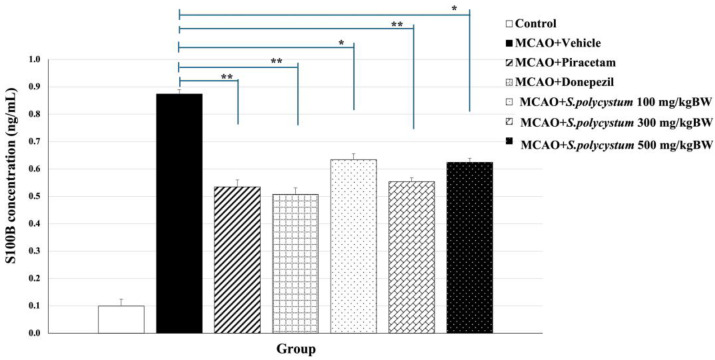
Effect of *S. polycystum* extract on a concentration of serum S100B. Each column and bar represent a mean value ± S.E.M. (n = 8/group). ** *p*-value < 0.01, * *p*-value < 0.05, compared to the vehicle group.

We investigated serum S100B levels, to determine the initial stages of mild cognitive impairment and the development of ischemic stroke. The effect of the extract on serum S100B concentration is shown in Figure 5. MCAO-exposed rats that received the vehicle showed a significant change in this parameter compared to control rats (*p* < 0.01). The *S. polycystum* extract at doses of 100, 300, and 500 mg/kg BW, decreased serum S100B levels compared to the vehicle group (*p* < 0.05, 0.01, and 0.05, respectively). Notably, we found that rats that received piracetam and donepezil had a significant reduction in the expression of serum S100B levels compared with the vehicle administration (*p* < 0.01 for both administrations).

The results, showing that administration of *S. polycystum* extract led to a reduction in serum S100B concentration, align with the hypothesis that a seaweed extract rich in phenolic compounds could enhance neuroprotective function, thereby inhibiting expression of S100B. These findings showed the extract’s ability to improve cognitive performance is contributed by its effects on reducing oxidative stress and exerting neuroprotective effects.

## 3. Discussion

During this study, we explored the phenolic content and antioxidant properties of nine types of seaweed, and aimed to select the greater seaweed species for developing functional ingredients to improve cognitive function in the middle cerebral artery occlusion (MCAO) stroke rat model. The candidate seaweed species included: *C. lentillifera*, *C. antennina*, *E. prolofera*, *S. polycystum*, *P. Minor*, *P. australis*, *G. pusillum*, *J. Rubens*, and *G. acerosa*, which were all extracted and analyzed for total phenolic content, radical scavenging activity, and reducing power. We also found that *S. polycystum*, which belongs to the Phaeophyta (brown seaweed) division, has a high amount of phenolic content and potent antioxidant activity. This is similar to the findings of Arsianti et al. [16] and Wu et al. [17], which reported that *S. polycystum* exhibited high total phenolic content, total flavonoid content, and antioxidant activity. Therefore, it appears that the *S. polycystum* extract may exhibit strong antioxidant properties, likely due to the phenolic compounds possessing significant antioxidant capabilities. Previous studies also suggest that phenolic compounds have significant antioxidant capabilities, anti-inflammatory activities, and neuroprotective effects, which help manage various ailments such as memory impairment [18,19]. Consequently, we evaluated the potential of *S. polycystum* extract, which is rich in phenolic content, for its neuroprotective and antioxidant effects in a MCAO rat model of ischemic stroke. Previous studies have reported that cerebral ischemia induced by the MCAO experimental model can disrupt blood flow or cause vascular changes, leading to insufficient blood supply to the brain, which can result in cognitive impairment [20].

Our data demonstrated that *S. polycystum* improved spatial memory by showing a decreased escape latency time and increased retention time in the Morris water maze test. Additionally, recognition memory, as evaluated by the novel object recognition test, increased. The cognitive enhancement effects of *S. polycystum* exhibited continuously before and after the MCAO operation throughout the study period. Therefore, *S. polycystum* extract has the potential to prevent and alleviate cognitive decline. These cognitive abilities are reported to be associated with the hippocampus, because it is an important structure responsible for cognitive functions, not only in episodic memory but also in processing speed, working memory, and executive function [21].

Based on accumulating evidence, oxidative stress plays a crucial role in the pathogenesis of cognitive impairment induced by ischemic stroke. Regarding in the study by Liu et al. [22], they found that MDA levels are associated with early cognitive impairment in patients with ischemic stroke and a previous study revealed the reduction in MDA concentration in the MCAO model with a significant decrease in infarct volume and the percentage of hemisphere infarct in the hippocampus area [23,24]. Alterations in the antioxidant enzyme activities were also reported [25,26]. Similarly, these changes were represented in this study, which showed that MCAO rats received a vehicle had different outcomes compared to control rats. Interestingly, our results revealed that all doses of *S. polycystum* extract decreased MDA levels and elevated activities of SOD, CAT and GSH-Px. Therefore, *S. polycystum* has the potential to exhibit the first-line defense through endogenous antioxidant enzymes, which are involved in the overall defense strategy against the generation of reactive oxygen species (ROS) resulting in a reduction of lipid peroxidation product in the hippocampus.

Over the last decade, recent studies have provided more evidence about the expression of S100B protein as a biomarker in the pathogenesis of central nervous system (CNS) injury and vascular cognitive impairment [27,28,29,30]. In this regard, increased levels of the S100B protein are stimulated by the expression of inflammatory cytokines, ROS, and apoptosis, through the contribution of astrocytes and microglia after brain injury [31]. This study has clearly demonstrated that the administration of *S. polycystum* extract for 35 days exhibited a reduction of serum S100B level. The result aligns with earlier studies suggesting that the reduction of S100B protein is achieved by reducing free radical damage and improving antioxidant enzymes through bioactive compounds such as epigallocatechin-3-gallate (EGCG) [32] and polyphenols, including those found in cinnamon [33] and green tea extract [34]. Therefore, *S. polycystum* might decrease serum S100B concentration by reducing oxidative stress and enhancing neuroprotective effects.

In addition, this study emphasized that *S. polycystum* extract contained a high amount of gallic acid, which is an important phenolic compound. Several scientific studies report that the neuroprotective effects of gallic acid alleviate cognitive deficits by improving cerebral antioxidant defenses [35] due to its capacity to cross the blood-brain barrier, directly reduce high levels of ROS and RNS, and chelate transition metal ions [13,36]. Moreover, gallic acid could modify the polarization of microglia and attenuate cerebral ischemia/re-perfusion-induced blood-brain barrier injury [37]. Thus, the possible underlying mechanism of *S. polycystum* extract in enhancing cognitive memory might involve the phenolic compounds, particularly gallic acid, which possesses antioxidant and neuroprotective activities as mentioned earlier. However, other bioactive compounds in *S. polycystum*, such as carotenoids, are also reported to regulate oxidative stress and neuroprotective activity [12,38]. These compounds may also contribute to the improved antioxidant activity, which in turn influences the overall effect of *S. polycystum*.

Based on those findings, we found that rats that received the extract at doses of 300 and 500 mg/kg BW showed significant improvement in cognitive performance. All doses demonstrated significant effects on antioxidative stress and S100B inhibition. However, it was found that the administration of *S. polycystum* showed no significant dose-dependent effects. This phenomenon may occur due to the crude extraction process. Additionally, extraction using ethanol solvent is suitable for extracting polar compounds and is very efficient in extracting phenolic compounds [39], resulting in a diverse range of compounds present in *S. polycystum* [17], which might contribute to these effects. Therefore, phenolics separation, identification, and quantification will be presented to clarify different phytochemicals, for further studies. Moreover, several studies have revealed that the presence of other bioactive compounds in *S. polycystum*, such as carotenoids and other polyphenols, could also contribute to the neuroprotective and antioxidant effects observed [40]. These compounds might interact synergistically or antagonistically, affecting the outcomes of the study. Thus, it is essential to critically assess the influence of these other bioactive compounds and to conduct further research to isolate and identify the active constituents contributing to the cognitive-enhancing effects.

While the study demonstrates the potential neuroprotective and antioxidant effects of *S. polycystum* extract in enhancing cognitive functions, there are certain limitations that need to be acknowledged. Firstly, the study did not explore the dose-dependence of the extract’s effects extensively. Although the results indicate significant improvements at various doses, there was no clear dose-response relationship observed. Another limitation is the use of an in vivo rat model for ischemic stroke-induced cognitive deficits. We did not evaluate the infarct size, the ability to achieve reperfusion, or the resultant penumbra area. Although the permanent middle cerebral artery occlusion model is one of the models that most closely simulates vascular dementia [41], the location and value of the infarct may influence cognitive impairments. Therefore, imaging and quantification methods, such as computed tomography (CT) and positron emission tomography (PET), should be performed in future studies to accurately determine infarct and penumbra volumes.

**Figure 6:**
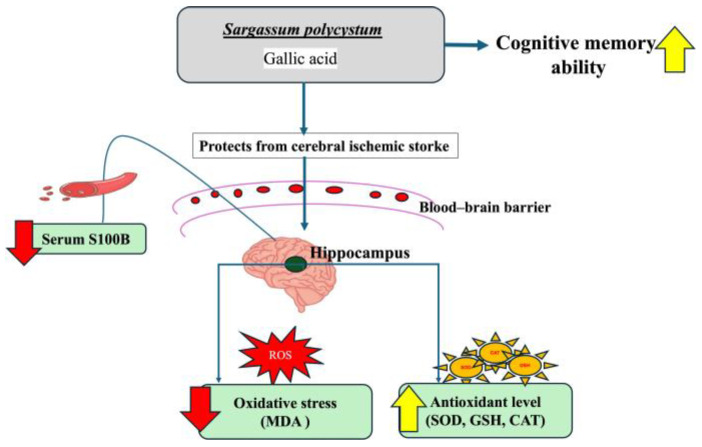
Graphical summary of the findings. The red arrow indicates a decrease in effect, while the yellow arrow indicates an increase in effect.

In summary, these outcomes suggest that the administration of *S. polycystum* extract, which contains phenolic compounds with both antioxidant and neuroprotective activities, may significantly contribute to improved cognitive function in a rat model of MCAO-induced ischemic stroke. A graphical summary of the findings is shown in Figure 6. However, further studies are required to confirm the findings of this study and to identify other currently unknown molecular mechanisms by which *S. polycystum* protects against cognitive impairment.

## 4. Materials and Methods

### 4.1. Chemicals and Reagents

Quercetin, gallic acid, and DPPH were procured from Sigma-Aldrich, purchased from Sigma-Aldrich Limited (St. Louis, MO, USA). All other chemical substances were of analytical grade and were purchased from Sigma Chemical Company (St. Louis, MO, USA, and Abcam, Cambridge, MA, USA).

### 4.2. Plant Material Preparation and Extraction

Fresh samples of nine seaweed types, including *Caulerpa lentillifera*, *Chaetomorpha antennina*, *Enteromorpha prolofera*, *Sargassum polycystum*, *Padina minor*, *Padina australis*, *Gelidium pusillum*, *Jania rubens*, and *Gelidiella acerosa*, were collected in Chon Buri, Thailand. The marine plants were identified by the Agricultural Scientist in the Botany and Plant Herbarium research group, Plant Varieties Protection Division.

After being cleaned, the fresh seaweeds were cut into small pieces and dried at 40 °C. All seaweeds were soaked with 80% ethanol solvent in water. After an overnight incubation, the supernatant was filtered with Whatman No.1 filter paper, and then the solvent was evaporated in rotary evaporator at 40 °C. The concentrated sample was stored at −20 °C until use.

### 4.3. Determination of Phenolic Contents

The phenolic content was observed using the Folin-Ciocalteu method, according to the study by Lee et al. [42]. Briefly, 0.5 mL of extract (100–1000 µg/mL) solution was mixed with 2.5 mL of Folin-Ciocalteau’s phenol reagent. After being placed in a dark room for 5 min, 2.0 mL of Na_2_CO_3_ was subsequently added to tube of the mixture and incubated for 2 h. UV spectrophotometer at 760 nm was used to evaluate the phenolic content. The phenolic content was quantified using a calibration curve of gallic acid, and finally, these results were expressed as milligrams of gallic acid equivalent per gram (mg GAE/g).

### 4.4. Determination of Antioxidant Activity Using DPPH Radical Scavenging Activity and Ferric Reducing Antioxidant Power (FRAP) Assay

The antioxidant activity of the crude extracts was determined using a slightly modified version of the DPPH method described by Yan-Hwa et al. [43]. 50 µL of crude extract solution (1–500 µg/mL) in methanol, and 100 µL of DPPH (0.2 mM) solution, were mixed and then placed in a dark room for 30 min. The absorbance at 517 nm was selected to measure the reaction. The radical scavenging activity (RSA) of DPPH was calculated against ascorbic acid, which was used as a positive control.

The FRAP assay was used to evaluate the antioxidant power of the extracts, which was assessed by measuring the reducing power of the extracts through the modified Fe^3+^ to Fe^2+^ reduction assay, as described by Hu et al. [44]. Briefly, 10 μL of the diluted seaweed extracts was mixed with 300 μL FRAP reagent. After incubation at room temperature for 30 min, the plate was read at a wavelength of 593 nm in UV spectrophotometer. Ascorbic acid (AA) was prepared as the standard. The reducing power of the extracts were calculated based on the standard curve and expressed as milligrams of ascorbic acid equivalent per gram (mg AAE/g).

### 4.5. Determination of Gallic Acid Content by HPLC Analysis

Gallic acid has beneficial activity against neurological ailments. Therefore, gallic acid content was evaluated using high-performance liquid chromatography (HPLC) (Agilent 1260 Series, Santa Clara, CA, USA). Chromatography of the gallic acid was conducted using a restek ultra C18 column (4.6 × 150 mm, 5 μm). The mobile phase was composed of water-acetoni-trile-acetic acid (88:10:2; *v*/*v*/*v*), and was delivered at a rate of 1 mL/min with a wavelength at 278 nm. Retention times and peaks area were recorded. An injection volume of 10 μL was used for the qualitative analysis of both the gallic acid standard and the extract. The peak areas corresponding to the extract were integrated with the gallic acid standards to calculate the precise amount of gallic acid contained in the extract.

### 4.6. Experimental Animals

Adult male Wistar rats weighing 180–220 g were used as experiment animals. They were purchased from the National Laboratory Animal Center, Salaya, Nakhon Pathom, Thailand. Animals were maintained on a 12:12 h light: dark cycle, and were provided with access and water *ad libitum*. The experimental protocols were approved by the Institutional Animal Care and Use Committee (IACUC), Burapha University, Thailand (No. 15/2565). After accommodation for 14 days, rats were randomly divided into various groups as the following

Group I: Naïve intact; rats in this group received no treatment.
Group II: Vehicle plus MCAO; rats in this group received a dimethyl sulfoxide (DMSO) solution, which was used as the vehicle treatment, 14 days before and 21 days after exposure to the occlusion of the right middle cerebral artery (Rt. MCAO).
Group III: Piracetam plus MCAO; rats in this group received piracetam (200 mg/kg BW), 14 days before and 21 days after exposure to Rt. MCAO.
Group IV: Donepezil plus MCAO; rats received donepezil (1 mg/kg BW), 14 days before and 21 days after exposure to Rt. MCAO.
Group V–VI: The selected extract plus MCAO; rats received *S. polycystum* extract (at doses of 100, 300, and 500 mg/kg BW), 14 before and 21 days after exposure to Rt. MCAO.

### 4.7. Animal Protocol

**Figure 7:**
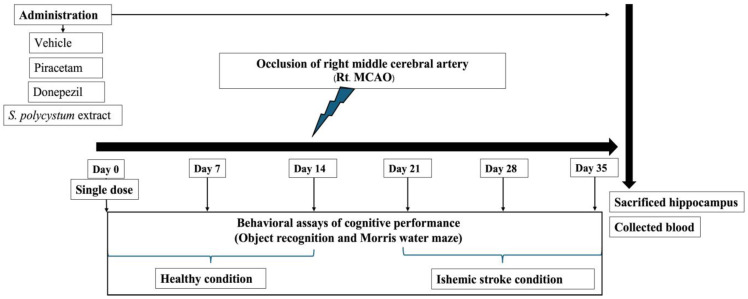
Flow chart of the experimental protocol of the animal study.

Rats were anesthetized with pentobarbital sodium at a dose of 50 mg/kg BW, before implementing the occlusion of the right middle cerebral artery, following the method described by Longa et al. [45]. Assigned substances were diluted in DMSO, and administered once daily for 14 days before and 21 days after exposure to Rt. MCAO. The battery of tests for assessing cognitive function, including Morris water maze and novel object recognition test, were performed throughout the study period. At the end of the study, on day 35, all animals were anesthetized with an intraperitoneal injection of sodium pentobarbital, at a dose of 100 mg/kg BW. The hippocampus was isolated to determine the oxidative stress status. Blood samples were collected at the end of the study, to evaluate S100B concentration. The flow chart of the experimental protocol of the animal study is shown in Figure 7.

### 4.8. Determination the Cognitive-Enhancing Effect

#### 4.8.1. The Morris Water Maze Test

The Morris water maze test is designed to assess spatial learning and memory. It measures the ability of rodents to navigate and remember the location of a hidden platform in a circular pool filled with water, relying on spatial cues provided in the environment. The test primarily assesses the function of the hippocampus, a brain region critical for spatial memory and navigation. Decreased escape latency (time taken to find the hidden platform) and increased retention time (time spent in the target quadrant during probe trials) indicate enhanced spatial learning and memory [46]. In the context of ischemic stroke model in this study, each rat was trained to locate and climb onto a platform, which was immersed in one quadrant of a circular tank (170 cm × 40 cm) filled with water covered by a non-toxic substance. On the day of the experiment (24 h later), rats were re-exposed to the same conditions, but the platform was removed from the quadrant in which it was previously located. The retention time and escape latency time were recorded individually for each rat before and after surgery.

#### 4.8.2. Novel Object Recognition Test (NOR)

The NOR test evaluates recognition memory by measuring the time spent exploring a novel object compared to a familiar one. This test is based on the natural tendency of rodents to spend more time exploring new objects in their environment. The recognition index is a useful measure of cognitive function, particularly related to the hippocampus and the medial prefrontal cortex. In our study, the NOR test was employed to determine the ability of *S. polycystum* extract to improve recognition memory in rats after ischemic stroke. An increase in the recognition index observed in treated groups suggests that the extract could enhance recognition memory [47]. This test was conducted in two phases: training and testing. In the training phase, individual rats were allowed to freely observe two identical objects, which were placed in an evaluation box, for 5 min. After a 10-min rest, the rats were transferred to the testing phase, where one of the two objects was replaced with a new object. The time each rat spent observing an object was recorded, and this was used to calculate the recognition index. The following equation was used to define the object recognition index:

Object Recognition Index = [TC / (TA + TC)] × 100

TA being the time the rat observed the initial object in the testing phase, and TC being the time the rat observed the new object in the testing phase.

### 4.9. Biochemical Assay

#### 4.9.1. Determination of Oxidative Stress Status

After anesthesia, the hippocampus was isolated and kept at a cool temperature in ice buckets, then homogenized in four volumes of 0.1 M phosphate buffer (pH 8.0). The supernatant was taken for this assessment. The levels of oxidative stress markers and the enzymatic antioxidant activities were determined to assess the oxidative stress status. MDA level was evaluated using the thiobarbituric acid reaction method [48], while SOD activity was assessed using an SOD assay kit (Cayman Chemical Company, Ann Arbor, MI, USA), based on the rate of reduction in the xanthine/xanthine oxidase system [49]. Superoxide dismutase from bovine liver (Sigma Chemical Company, St. Louis, MO, USA) was used to prepare the standard curve, and calculate the SOD activity of each sample.

CAT activity was determined by measuring the rate of H_2_O_2_ degradation. The reagent mixture, containing 50 mM of potassium permanganate (KMnO_4_), 5N H_2_SO_4_ and 0.1 M H_2_O_2_ was prepared at room temperature in the dark. Then, the supernatant sample and all reagents were mixed. The absorbance was recorded at 515 nm. Catalase from bovine liver (Sigma, USA) was used to construct the standard curve and calculate the CAT activity of each sample [50].

The GSH-Px activity was determined as previously described [51]. The absorbance was recorded at 314 nm using a spectrophotometer. Glutathione peroxidase from bovine erythrocytes (Sigma, USA) was used to construct the standard curve and calculate GSH-Px activity.

#### 4.9.2. Determination of S100B Serum Level

Blood samples were collected from rats at the end of the study period. The samples were then centrifuged to separate plasma and serum. Serum S100B levels were determined by enzyme-linked immunosorbent assay (ELISA) using a commercially available kit (LSBio, An Absolute Biotech Company, Seattle, WA, USA). According to the instructions of an assay kit, all samples were incubated with a biotin-labeled monoclonal antihuman S100B antibody, followed by the addition of the secondary antibody to the wells. After stopping reaction, the optical density (OD) was measured spectrophotometrically at a wavelength of 450 nm.

### 4.10. Statistical Analysis

Data are presented as the means ± S.E.M. and were statistically analyzed using one-way ANOVA, followed by a post hoc test using SPSS version 13. The result was considered statistically significant if the *p*-value was less than 0.05.

## 5. Conclusions

Based on the results from the present study, the *S. polycystum* extract, a novel functional ingredient, ameliorates cognitive dysfunction. The possible underlying mechanism may be to improve the antioxidant activity and neuroprotective effect in the hippocampus, which in turn enhances cognitive memory ability. Furthermore, the results emphasized that the *S. polycystum* extract, which contained phenolic compounds, particularly gallic acid, may offer significant benefits and be more suitable for patients with cognitive impairment. However, future research should include studies that aim to isolate and characterize individual bioactive compounds within *S. polycystum* extract to ensure their specific contributions to neuroprotection. Additionally, dose-response studies, long-term safety evaluations, and clinical trials in human subjects will be crucial to validate the findings and establish the therapeutic potential of *S. polycystum* extract.

## Figures and Tables

**Table 1:** Total phenolic content, antioxidant activity, and reducing power of selected seaweed extracts. GAE: gallic acid equivalents; AAE: ascorbic acid equivalents.

Division of Seaweed, Species	Total Phenolic Content (mg GAE/g Dry Extract)	Radical Scavenging Activity of DPPH (µg/mL)	Reducing Power (mg AAE/g Extract)
Chiorophyta, *Caulerpa lentillifera*	3.42 ± 0.25	32.71 ± 0.43	9.56 ± 4.23
Chiorophyta, *Chaetomorpha antenina*	16.42 ± 1.21	45.26 ± 3.51	15.00 ± 0.84
Chiorophyta, *Enteromorpha prolofera*	32.72 ± 2.54	82 ± 2.47	63.00 ± 6.92
Phaeophyta, *Sargassum polycystum*	92.25 ± 3.26	124.36 ± 5.04	63.24 ± 2.79
Phaeophyta, *Padina Minor*	54.27 ± 1.59	205.32 ± 2.01	34.52 ± 3.42
Phaeophyta, *Padina australis*	76.29 ± 2.42	159.42 ± 1.83	35.82 ± 2.54
Rhodophyta, *Gelidium pusillum*	15.95 ± 1.38	114.25 ± 3.24	29.45 ± 3.52
Rhodophyta, *Jania Rubens*	22.47 ± 1.05	62.42 ± 4.27	12.08 ± 2.54
Rhodophyta, *Gelidiella acerosa*	32.98 ± 1.32	56.42 ± 5.62	16.34 ± 3.02

Values represent the mean ± standard deviation of three independent investigations.

## Data Availability

The data sets used and/or analyzed during the current study are available from the corresponding author upon reasonable request.
